# Clinical and Optical Coherence Tomography Characteristics of Severe Intraretinal Silicone Oil Migration

**DOI:** 10.1177/24741264251356293

**Published:** 2025-08-05

**Authors:** Carl Shen, Daisy Liu, Chao Chen, Sterling King, Mark Greve, David Ehmann, Mark Seamone

**Affiliations:** 1 Department of Ophthalmology and Visual Sciences, University of Alberta, Calgary, AB, Canada

**Keywords:** intraretinal silicone oil, retinal detachment, ILM peeling

## Abstract

**Purpose:** To characterize the clinical and imaging features of patients presenting with a constellation of findings believed to represent severe intraretinal migration of silicone oil. **Methods:** Query of our institutional electronic medical record system for patients who underwent silicone oil removal. **Results:** Twenty-eight cases of severe IRSO were identified. All patients had characteristic round hypo-reflective spaces favoring the inner retinal layers on OCT with increased hyperreflectivity in the deeper retinal layers, and corresponding hyperreflective spherules on near-infrared enface imaging. All patients received 5000 centistoke silicone oil and had had their macular internal limiting membrane removed by the time of final silicone oil instillation. Oil was in situ for an average 220 ± 198 days before removal. Average follow up from time of oil out was 353 ± 529 days. **Conclusions:** We characterize an underrecognized phenotype of intraretinal silicone oil which presents with a constellation of OCT findings distinct from other consequences of silicone oil use.

## Introduction

Silicone oil (SO) is commonly used as an intraocular tamponade for the treatment of complex retinal detachments (RDs), including giant retinal tears, inferior RDs, diabetic tractional RD, and RD with proliferative vitreoretinopathy (PVR).^1–3^

Despite being commonly used, several complications associated with SO tamponade have been reported, including migration into ocular tissue.^2,4^ Histopathological reports have found SO droplets in the cornea, iris, ciliary body, trabecular meshwork, retina, choroid, and optic nerve of enucleated eyes filled with SO.^5,6^ However, the characterization of this phenomenon in vivo in humans has been sparse. Intraretinal SO vacuoles are evident as early as 1 week in rabbit models and have been detected 2 months after injection in human enucleated eyes.^5,7^ The mechanisms underlying intraretinal SO migration are not yet fully understood, but several factors have been associated with its development, including the oil’s viscosity, the presence of retinal breaks, and the duration of tamponade.^4^

Previous reports of intraretinal SO migration detected by optical coherence tomography (OCT) have largely described hyperreflective foci in the outer retina and optic nerve.^4,8–11^ We noted several patients presenting to our clinic with poor vision after SO removal had a pattern of OCT findings unlike the previously reported hyperreflective phenotypes. We endeavored to characterize this phenotype by identifying patients with severe cases of intraretinal SO migration and presenting their clinical and OCT imaging features.

## Methods

This retrospective consecutive case series was approved by the University of Alberta research ethics board (Pro00121465). The electronic medical records (HealthQuest) were searched to identify all operative reports for cases of SO removal between November 1, 2016, and June 2, 2022. All types of rhegmatogenous RD, including eyes with primary PVR, giant retinal tear, retinoschisis, or macular hole (MH) were included. Patients were excluded if they had inadequate image quality, less than 1 month of follow-up after SO removal, or SO was still in situ at last follow-up. We defined the presence of intraretinal SO migration by the criteria outlined in the Supplemental Material. Severe criteria included features felt to most likely result in functional deficits, specifically location and total quantity of intraretinal SO migration. Severe cases were defined by the presence of more than 50 droplets of intraretinal SO on the Early Treatment Diabetic Retinopathy Study (ETDRS) macular grid and involvement of the central subfield. All cases were reviewed by 2 authors (C.S., D.L.).

Data collected included age, sex, laterality, presenting visual acuity (VA) and macular status, presence of preexisting ocular disease, RD characteristics, specifics of surgical repair, clinical and imaging characteristics while SO was in situ, postoperative complications, and final VA (Table 1). Final outcome data were collected up to September 15, 2022. Snellen VA was converted to logMAR for statistical analysis and converted back to Snellen for data presentation. Count fingers and hand motion vision was converted to logMAR notation, as previously described.^12^ Light perception (LP) was not included in statistical analyses but was described qualitatively. Statistical analysis using basic descriptive statistics was performed with Excel software (Microsoft).

**Table 1.** Patient Demographics (N = 28).

Demographic	Value
Sex (n)	
Female	10
Male	18
Age (y)	
Mean ± SD	66.5 ± 16
Range	19, 92
Laterality	
Left eye	17
Right eye	11
Presenting VA	
Mean ± SD	20/400 ± 205
Range	30, LP
CF	4
HM	7
LP	2
Not recorded	1
Previous surgical history	
Cataract surgery	20
Pars plana vitrectomy	12
Scleral buckling	5
Strabismus	1
Globe rupture repair	1
LASIK	2

Abbreviations: CF, counting fingers; HM, hand motions; LASIK, laser in situ keratomileusis; LP, light perception.

All surgeries were performed by 1 of 8 vitreoretina surgeons in a group retinal practice in Edmonton, Alberta, Canada as previously described.^13^ Retinal tamponade was done in all cases with 5000 centistoke SO (Oxane 5700, Bausch + Lomb) after fluid–air exchange. The concurrent addition of phacoemulsification or a scleral buckle was at the surgeon’s discretion. A standard 3-port, 23-gauge vitrectomy setup (Constellation 5000 Vision System, Alcon Inc) with a single valveless trocar was used for SO removal, the bulk of which was removed by active suction using a blue tip oil extractor under viscous fluid control (VFC Pack, Alcon Inc). Multiple fluid–air exchanges were performed until SO was no longer visible. Concurrent anterior chamber washout, cataract surgery, membrane peeling, laser retinopexy, scleral buckling, and final tamponade (fluid, air, sulfur hexafluoride, perfluoropropane) were left to the surgeon’s discretion at the time of SO removal.

## Results

**Figure 1.**
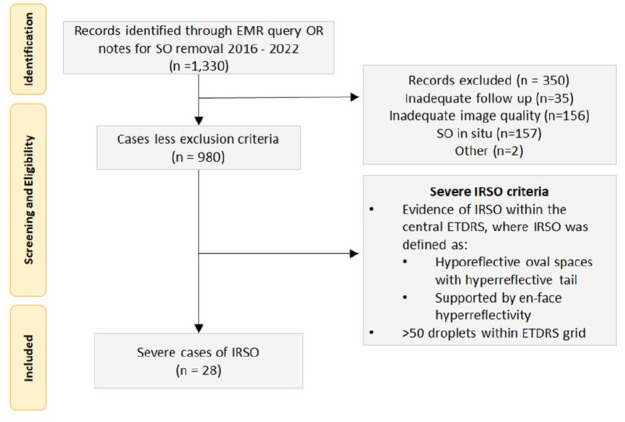
Inclusion and exclusion criteria.

**Figure 2.**
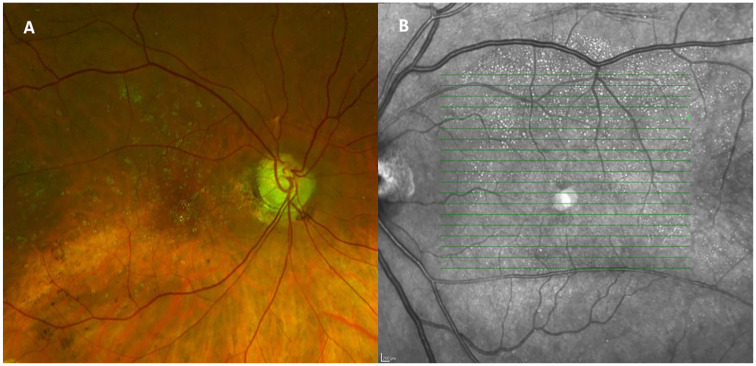
(A) Color reconstruction fundus photography shows intraretinal silicone oil (SO) migration as yellow spherules with a clear central cavity in the macula. (B) Near-infrared en face imaging shows intraretinal SO migration as hyperreflective circles of varying sizes, favoring the superior macula. The areas appear more distinct than on fundus photography.

**Figure 3.**
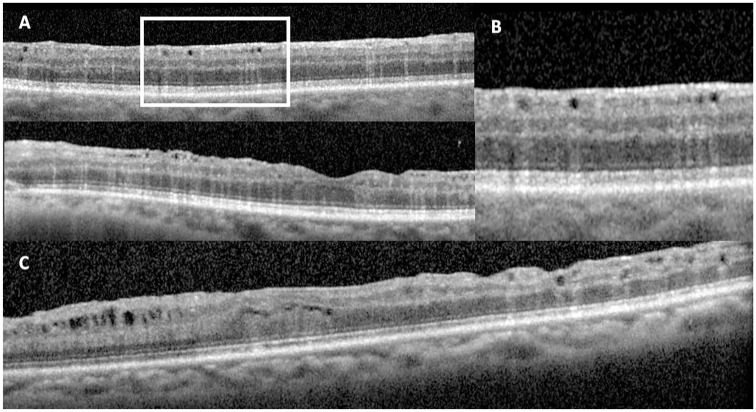
(A) Optical coherence tomography (OCT) images of intraretinal silicone oil (SO) migration in the superior macula (top) and through the fovea (bottom) shows hyporeflective spaces that correspond with the en face imaging with hyperreflective streaks. These spaces tend to favor the inner retina and are randomly distributed. (B) Magnification of (A) highlights the vertical hyperreflective streaks deep to the hyporeflective cavities, a useful feature to distinguish intraretinal SO migration from other causes of cystic spaces in the retina. (C) OCT image shows cystoid macular edema/microcystic edema (left) and organized, hyporeflective spaces without hyperreflective streaks and intraretinal SO migration (right).

In total, 1300 eyes underwent SO removal between November 1, 2016, and June 2, 2022. Three hundred and fifty-one cases were excluded, 35 cases were lost to follow-up or were followed elsewhere after SO removal, 157 cases due to SO remaining in situ at the time of final follow-up (oil exchanged at time of SO removal or subsequent surgery with SO tamponade remaining in situ), and 156 cases due to inadequate OCT image quality (Figure 1). Of the 980 cases deemed adequate for assessment, 28 cases (2.9%) met the criteria for severe intraretinal SO migration and were included in this series. Illustrative fundus photographs and near-infrared images are shown in Figure 2. All patients had evidence of hyporeflective oval spaces favoring the inner retinal layers, with a hyperreflective tail of the deeper retinal layers particularly noticeable in the outer nuclear layer (Figure 3). These vacuoles corresponded with hyperreflective foci on en face imaging and averaged approximately 50 µm in width. In all cases, the intraretinal SO particles were greater in number in the superior half of the macular ETDRS grid than the inferior half.

The indication for SO placement in all cases was RD. Twenty (71%) of the cases were primary detachments, and 8 (29%) were re-detachments. Of the 20 primary detachments, 14 (70%) were PVR detachments and 1 (5%) was a giant retinal tear. Vitrectomy had been performed in 12 patients by final SO instillation, 5 of which had received previous SO instillation. Notably, all patients in this series had the macular internal limiting membrane (ILM) removed before final SO instillation. Additional procedures performed at time of SO instillation included cataract extraction in 4 cases, scleral buckling in 9 cases, and cataract extraction and scleral buckling in 1 case (Table 2).

**Table 2.** Surgical Characteristics of Patients With Severe Intraretinal Silicone Oil (N = 28).

Characteristic	Finding
Surgery at final oil in	
PPV	14
PPV/CE	4
PPV/CE/buckle	1
PPV/buckle	9
Surgical indication for oil	
RD	20
Recurrent	8
Fovea status at presentation	
Off	22
On	6
Quadrants involved	
1	4
2	13
3	6
4	4
Additional RD characteristics	
PVR	21
Previous PPV	12
Previous SO	5
Macular hole	1
Giant retinal tear	1
Lens status at oil in	
Pseudophakic	20
Phakic	8
ILM peeled	
Before surgery to final oil in	
Yes	6
No	22
At time of final oil in	
Yes	28
No	0
Tamponade at time of oil out	Fluid: 9/28
	Air: 6/16
	SF6: 11/28
	C3F8: 2/28
Buckle at time of oil out:	No: 20/28
	Yes: 8/28

PPV = Pars Plana Vitrectomy, CE = Cataract Extraction.

SO.

ILM.

C_3_F_8_, perfluoropropane.

SF_6_, sulfur hexafluoride.

Proliferative Vitreoretinopathy.

Retinal Detachment.

**Figure 4.**
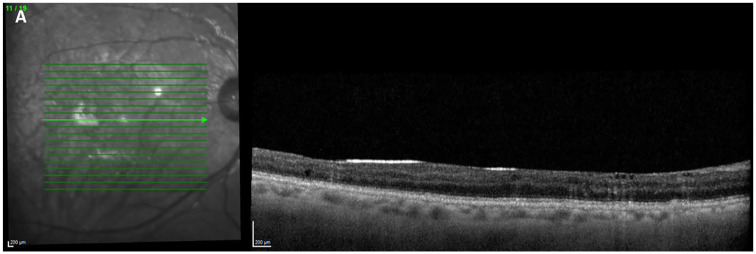
Optical coherence tomography image shows the development of intraretinal silicone oil (SO) migration with the SO still in situ.

SO remained in situ for an average of 220 ± 198 days (range, 87-935) (Table 3). OCT features of intraretinal SO migration were detectable in 20 of 23 (87%) cases while SO was in situ (Figure 4). The 5 cases with multiple SO instillations were not included in this assessment. These features were first detectable as early as 16 days after SO instillation and as late as 523 days while SO was in situ. The highest measured intraocular pressure (IOP) while SO was in situ was on average 20.5 mm Hg ± 7 mm Hg (range, 11-38). Only 1 case of prolonged IOP elevation requiring glaucoma surgery was noted in the period after SO removal and none while SO was in situ.

**Table 3.** Characteristics With SO In Situ (N = 23).

Parameter	Value
Duration of oil in situ (d)	
Mean ± SD	220 ± 198
Range	87, 935
Intraretinal SO detectable while oil in situ, n (%)	20 (87)
Time from oil in to earliest Intraretinal SO detectable in situ (d)	
Mean ± SD	70 ± 113
Range	16, 523
Highest IOP with oil in situ (mm Hg)	
Mean ± SD	20.5 ± 7
Range	11, 38

Abbreviations: IOP, intraocular pressure; SO, silicone oil.

The average presenting VA was 20/400 ± 205 with 2 patients presenting with LP-only vision (Table 4). The best documented VA while SO was in situ was 20/160 ± 44 at 58 ± 147 days. The final VA was 20/200 ± 57 at 353 ± 529 days after SO removal. All patients were pseudophakic at the time of final VA assessment except for 1 patient, who was phakic. Compared with the initial presenting VA, 16 of 27 (60%) patients improved, 7 (26%) patients worsened, 4 (15%) remained the same, and 1 patient did not have a presenting VA recorded. Compared with the best VA achieved while SO was in situ, the final VA was the same or improved after SO removal in 10 of 28 (36%) cases and worse in 18 (64%) cases. In the 4 patients who presented with macula-on RDs without subsequent recurrent detachment, the presenting VA was 20/30, 20/40, 20/80, and 20/100. The final VA in these patients after SO removal was 20/200, 20/400, 20/80, and 20/150, respectively. Complications in the postoperative follow-up period after SO removal included cystoid macular edema (CME) in 14 of 28 (50%) cases, high IOP/glaucoma in 5 (18%), and MH development in 2 (7%) cases. Average central macular thickness at final follow-up was 279 ± 46 µm.

**Table 4.** Visual Acuity of Patients With Severe Intraretinal Silicone Oil (N = 28).

Parameter	Value
Presenting VA	
Mean ± SD	20/400 ± 8911
Range	30, LP
CF (n)	4
HM (n)	7
LP (n)	2
Not recorded (n)	1
VA before oil out	
Mean ± SD	20/400 ± 70
Range	60, 2000
Time from oil in to VA before oil out (d)	
Mean ± SD	114 ± 176
Range	61, 734
Best VA oil in situ	
Mean ± SD	20/160 ± 44
Range	40, 800
Time from oil in to best VA oil in situ (d)	
Mean ± SD	58 ± 147
Range	1, 734
Final follow-up from oil out (d)	
Mean ± SD	353 ± 529
Range	63, 2000
Final VA	
Mean ± SD	20/200 ± 57
Range	80, 2000
Lens status final VA (n)	
Pseudophakic	27
Phakic	1
Final VA better than best VA oil in situ (n)	
Yes	10
No	18

Abbreviations: CF, counting fingers; HM, hand motions; LP, light perception; VA, visual acuity.

## Conclusions

Several OCT phenotypes relating to SO tamponade have been described in the literature, including inner nuclear layer microcysts,^14^ retinal thinning,^15^ and hyperreflective emulsified silicone–retina interfaces.^16,17^ Intraretinal SO migration has been characterized primarily as intraretinal and subretinal small hyperreflective foci. OCT of the anterior segment was used by Errera et al^4^ to describe mechanically emulsified SO seen in model eyes. We characterized a distinct phenotype of circular hyporeflective spaces, presumably SO droplets, found chiefly in the inner retina, with an increased hyperreflective tail deep to the droplets and corresponding hyperreflective spherules on near-infrared en face imaging.

Two previous studies have reported on a similar phenotype as our series. Using time-domain OCT, Chung and Spaide^18^ described a single case of hyporeflective vacuoles, presumed to be intraretinal SO, after MH surgery. Although in that case the red-free photograph appears comparable to our fundus photographs, the hyporeflective vacuoles on OCT were significantly larger and wider and lacked deeper hyperreflectivity. A direct comparison of the cases is limited by the resolution of Chung and Spaide’s time-domain imaging. In a commentary to the seminal Errera et al^4^ study, Yu and Fisher^19^ acknowledge 3 different manifestations of intraretinal SO migration on OCT, including clear bubbles with/without hyperreflective tails, hyperreflective dots with/without hyperreflective tails, and hyperreflective tails without observable dots or bubbles. However, further characterization of these entities has not been published. We hypothesize that the hyperreflective tails represent an optical lensing phenomenon from the small oil droplets. The incidence of CME with SO tamponade has been reported to be between 14% and 36%.^20^ Significant heterogeneity among these studies exists, including the duration of tamponade, type of SO used, and different inclusion criteria for type of detachment. The rate of CME in our study of 50% was notably higher, which may reflect a true increased propensity to developing CME in cases of severe intraretinal SO migration related to associated inflammatory insults. However, with the multiple phenotypes of intraretinal SO migration described,^4,18,19^ including those without unique hyperreflective tails and near-infrared imaging features, it may not be possible to definitively distinguish the presence of CME from intraretinal SO migration in patients with multiple cystic spaces evident on OCT. This may lead to an inaccurate characterization of CME or the other phenotypes of intraretinal SO migration not described in the current study.

The cause for the variation in OCT phenotypes reported in our and previous studies is unclear. The difference may be accounted for by the physical properties of the SO. All patients in our series received 5000 centistoke SO, whereas previous reports of intraretinal SO migration have been mostly patients who received 1000 centistoke SO.^8,10,11^ The higher surface tension of 5000 centistoke SO may theoretically resist emulsification but results in larger droplets when emulsified.^21,22^ Although SO emulsification is a well-established precursor to other complications, such as glaucoma, the relationship between emulsification and intraretinal SO migration is not well defined. By definition, intraretinal SO migration could be considered emulsified SO because the droplets remain as distinct entities from the primary SO bubble. However, several observations from our cohort would suggest emulsification as we traditionally recognize it may not be a necessary precursor to the development of intraretinal SO migration.

Clinically, emulsified SO in the posterior segment appears as strongly reflective granular particles suspended throughout the vitreous or as patches of fine SO bubbles in hyperreflective collections on the surface of the retina. No clinical or OCT observations of the latter were appreciated in any of our patients while SO was in situ or during removal, which one would expect in the areas of intraretinal SO migration if they were a result of traditional emulsification. Rather, the distribution of SO particles in our cohort is in a diffuse manner in the posterior pole, favoring the superior macula. The propensity for the superior macula suggests a component of mechanical factors related to the buoyant force of the SO contributing to the development of intraretinal SO migration. In an upright position, the SO bubble will exert a relatively greater force on the superior macula than the inferior. This buoyancy may contribute to a transudative process of the primary SO bubble into the retina. Notably, evidence of intraretinal SO migration was only evident in areas of peeled ILM. Histological studies^5,23–25^ have identified intraretinal SO migration in both the extracellular matrix as well as within macrophages in the retina. The macrophage localization and engulfment are felt to be related to a local inflammatory response rather than an active engulfment that draws the SO into the retina.^23^

Previous studies have acknowledged the importance of ILM removal as a predisposing feature to the development of intraretinal SO migration.^11,18^ Intuitively, the ILM may act as a natural barrier to the penetration of SO irrespective of the mechanism. Interestingly, previous reports of the fine hyperreflective foci of intraretinal SO migration have been recognized in patients with an intact ILM,^9^ with only 26% of patients with intraretinal SO migration having had the ILM removed. Yet, to develop the severe phenotype of intraretinal SO migration we are observing, ILM removal appears to be a prerequisite.

Vision loss after SO tamponade has been reported in about 25% of cases; however, the cause of this vision loss in a significant subset of cases remains unknown.^26^ A subset of these patients may represent individuals with vision loss due to migration of SO into the retina. Because visual outcomes are often poor in complex RDs, it is difficult to draw conclusions from the VA data in our cohort. Nevertheless, several features in our patients suggest a detrimental effect from the presence of severe intraretinal SO migration. The peak best-corrected VA (BCVA) (20/160 ± 44) while SO was in situ was obtained on average at 2 months, and the average BCVA at the time of SO removal on average at 4 months was 20/400 ± 70. After SO removal, 18 of 28 (64%) patients did not have a final visual outcome better than their peak BCVA under SO. Of the 4 cases of macula-on RD repaired with a single surgery, 1 patient maintained their presenting VA, and the 3 others lost vision at final follow-up after SO removal. We are currently performing a study of all cases of complex RD with SO with and without the findings of intraretinal SO migration to better address the question of the functional effects on BCVA and other outcomes with a control group.

In our cohort, the average duration of SO tamponade before removal was 7 months. In the publicly funded Canadian healthcare system, with limited access to operating room resources, SO removal cases are usually the first to be postponed and largely viewed as elective. The optimal timing of SO removal is not definitively established.^27–33^ The theoretical increase in potential complications related to SO tamponade with time must be balanced against the need for prolonged tamponade to ensure anatomic stability of the retina. The phenomenon of severe intraretinal SO migration may be an additional complication to consider in the evaluation of timing of SO removal. A peak BCVA at 2 months after SO placement, and the fact that no patient had SO removal less than 3 months, may suggest a benefit to earlier SO removal in avoiding intraretinal SO. At the same time, the earliest noted intraretinal SO migration in our cohort was at the second postoperative visit, 16 days after SO placement, suggesting this phenomenon may begin shortly after SO placement in some cases and a safe time period for avoiding intraretinal SO may not exist. Further study on the clinical implications of severe intraretinal SO migration will better define the weight that should be placed on this observation.

Several limitations to our study exist. No histological correlates were available in our cohort to definitively confirm the presence of SO in the retina. We believe the constellation of hyporeflective spaces, with a deep hyperreflective tail and hyperreflectivity on en face imaging, was sufficiently specific to distinguish these intraretinal SO lesions from similar entities, such as CME and atrophic cystic spaces (Figure 3). We did not formally evaluate the presence of the hyperreflective foci phenotype in our cohort of patients because it was thought this finding could not be reliably specific to intraretinal SO migration against other hyperreflective constituents (retinal pigment epithelium cells, blood, inflammatory cells, etc). Due to the retrospective nature of the study, standardized documentation of emulsification was not done and thus the relationship between emulsification and intraretinal SO migration could not be studied. In addition, no grading schema for severity of intraretinal SO exists. In our study, severe disease was designated as more than 50 droplets within the macular EDTRS grid and involvement of the foveal subfield, which helped characterize our observations of this phenotype. Future studies will benefit from a standardized grading system.

In conclusion, we describe a relatively novel phenotype of intraretinal SO migration and the associated OCT imaging characteristics that distinguish it from similar intraretinal cystic spaces and previously described phenotypes. We confirm the necessity of ILM removal to allow for severe intraretinal SO migration. The clinical implications of severe intraretinal SO migration are not yet entirely clear; however, a deleterious effect is suggested by the poor visual outcomes in our cohort and the loss of BCVA from the time of SO in situ to final follow-up in many cases. As such, two cautious actionable steps may be taken in cases of complex RD in which SO is used as tamponade. First, the necessity for ILM peeling of the macula should be assessed on a case-by-case basis and, when warranted, foveal-sparing peeling may be considered. Second, because intraretinal SO migration can be detected with SO still in situ, monitoring for this finding may guide the optimal timing of SO removal against the need for prolonged tamponade, allowing for successful RD repair.

## Supplemental Material

sj-tif-1-vrd-10.1177_24741264251356293 – Supplemental material for Clinical and Optical Coherence Tomography Characteristics of Severe Intraretinal Silicone Oil Migration Supplemental material, sj-tif-1-vrd-10.1177_24741264251356293 for Clinical and Optical Coherence Tomography Characteristics of Severe Intraretinal Silicone Oil Migration by Carl Shen, Daisy Liu, Chao Chen, Sterling King, Mark Greve, David Ehmann and Mark Seamone in Journal of VitreoRetinal Diseases
