# Supplementary figures and images for: Clinical and Optical Coherence Tomography Characteristics of Severe Intraretinal Silicone Oil Migration
Source: J Vitreoretin Dis. 2025 Aug 5:24741264251356293. Online ahead of print. doi: 10.1177/24741264251356293 (PMC12328352; doi:10.1177/24741264251356293)

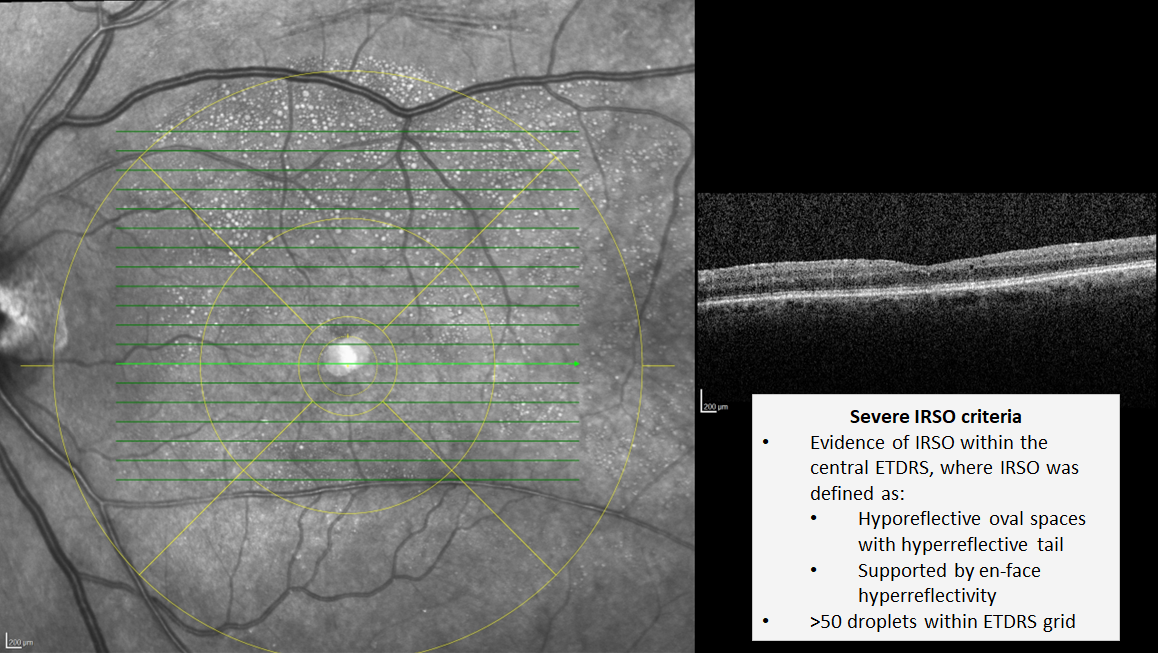

Supplement: sj-tif-1-vrd-10.1177_24741264251356293 – Supplemental material for Clinical and Optical Coherence Tomography Characteristics of Severe Intraretinal Silicone Oil Migration [file sj-tif-1-vrd-10.1177_24741264251356293.tif]
